# Integration of Serum Protein Biomarker and Tumor Associated Autoantibody Expression Data Increases the Ability of a Blood-Based Proteomic Assay to Identify Breast Cancer

**DOI:** 10.1371/journal.pone.0157692

**Published:** 2016-08-10

**Authors:** Meredith C. Henderson, Alan B. Hollingsworth, Kelly Gordon, Michael Silver, Rao Mulpuri, Elias Letsios, David E. Reese

**Affiliations:** 1 Provista Diagnostics, Inc., 55 Broad Street, 18^th^ floor, New York, NY, United States of America, 10004; 2 Mercy Women’s Center-Oklahoma City, 4300 McAuley Boulevard, Oklahoma City, OK, United States of America, 73120–8302; University of North Carolina School of Medicine, UNITED STATES

## Abstract

Despite significant advances in breast imaging, the ability to accurately detect Breast Cancer (BC) remains a challenge. With the discovery of key biomarkers and protein signatures for BC, proteomic technologies are currently poised to serve as an ideal diagnostic adjunct to imaging. Research studies have shown that breast tumors are associated with systemic changes in levels of both serum protein biomarkers (SPB) and tumor associated autoantibodies (TAAb). However, the independent contribution of SPB and TAAb expression data for identifying BC relative to a combinatorial SPB and TAAb approach has not been fully investigated. This study evaluates these contributions using a retrospective cohort of pre-biopsy serum samples with known clinical outcomes collected from a single site, thus minimizing potential site-to-site variation and enabling direct assessment of SPB and TAAb contributions to identify BC. All serum samples (n = 210) were collected prior to biopsy. These specimens were obtained from 18 participants with no evidence of breast disease (ND), 92 participants diagnosed with Benign Breast Disease (BBD) and 100 participants diagnosed with BC, including DCIS. All BBD and BC diagnoses were based on pathology results from biopsy. Statistical models were developed to differentiate BC from non-BC (i.e., BBD and ND) using expression data from SPB alone, TAAb alone, and a combination of SPB and TAAb. When SPB data was independently used for modeling, clinical sensitivity and specificity for detection of BC were 74.7% and 77.0%, respectively. When TAAb data was independently used, clinical sensitivity and specificity for detection of BC were 72.2% and 70.8%, respectively. When modeling integrated data from both SPB and TAAb, the clinical sensitivity and specificity for detection of BC improved to 81.0% and 78.8%, respectively. These data demonstrate the benefit of the integration of SPB and TAAb data and strongly support the further development of combinatorial proteomic approaches for detecting BC.

## Introduction

Breast cancer (BC) is the most commonly diagnosed malignancy and is the leading cause of cancer mortality among women [1]. Approximately 40,000 deaths from the disease occur annually in the US [2]. Detection of early-stage BC is widely recognized as being associated with a high cure rate and less morbid treatment. Unfortunately, even after decades of widespread mammographic screening, the rate at which women present at a later stage of BC has been only marginally reduced [3–5]. Multi-modality screening (using whole breast ultrasound or breast magnetic resonance imaging, MRI) has demonstrated significant improvement in cancer detection [6], but these approaches are limited to a minority of patients who are at high risk and/or have high mammographic density, with additional restrictions dictated by cost and feasibility. Furthermore, critics have pointed out that multi-modality screening will increase the number of unnecessary biopsies and could also add to the issue of over-diagnosis [7–9].

The American College of Radiology adopted BI-RADS^®^ (Breast Imaging–Reporting and Data System) as a quality assurance approach to standardize the lexicon used in breast imaging reports, as well as affording the opportunity to monitor outcomes and to update the system as new information accumulates [10]. Specifically, each BI-RADS category is associated with a probability of breast cancer–Level 1 (negative), Level 2 (benign finding), Level 3 (probable benign finding, below 2% chance of malignancy), Level 4 (suspicious finding, 2–94% probability of malignancy), and Level 5 (highly suspicious finding, > 95% probability of malignancy) [11]. It is important to note that, despite a seemingly simple classification system, misread mammograms account for up to 75% of malpractice claims against radiologists [12].

The primary decision point for breast cancer radiologists is between the 3 and 4 assessment. In BI-RADS 4 cases, the radiologist will recommend biopsy. In BI-RADS 3 cases, they likely will not. It is important to note that Category 4 includes a wide range of probabilities between Category 3 and 5, leaving the positive predictive value (PPV) in a wide range for Category 4 and thus somewhat imprecise [13,14]. Category 4 was later sub-divided into 4a (low index of suspicion for malignancy), 4b (intermediate suspicion), and 4c (moderate suspicion, but not conclusive). Yet the current standard of care is to biopsy all Category 4 and 5 lesions, so while the sub-division of Category 4 improves quality assurance monitoring, the relatively low PPV remains unchanged overall. Improving upon the Category 4 PPV would be a welcome addition, especially for those concerned with the harms of screening.

In clinical practice, a NPV would provide a superior level of confidence when determining whether to re-assess a patient at 6 months or follow with more aggressive screening at the current visit. In total, improving PPV could help “rule in” biopsy for some BI-RADS 3 lesions and BI-RADS 4a where the radiologist is sometimes faced with a subjective decision about whether or not to perform biopsy. The standard of care (SOC) for BI-RADS 3 lesions is six month follow-up imaging, but patient compliance with this recommendation can be problematic [15]. While a BI-RADS 3 lesion may have only a 2% chance of malignancy, if that patient does not return for the six month follow-up study the chance for early diagnosis could be lost. At the same time, improving the negative predictive value (NPV) could assist radiologists in the decision to “rule out” biopsy in this same patient population.

Contrarily, women who undergo biopsy for a false-positive breast lesion may experience a range of post-procedure effects, from future false-positive mammograms to rare complications related to invasive biopsies [16,17]. In the US, mammogram guidelines proposed by the American Cancer Society [18] have recently changed from beginning annual exams at age 40 to age 45, due in part to the large number of false-positive findings in younger women [19]. Furthermore, a recent UK study noted that women who were told that their mammogram findings were likely false-positive wanted alternatives to invasive biopsy, including additional imaging and watchful waiting [20]. Serum-based biomarkers may provide some relief from these concerns as they are based on an additional objective measurement; therefore, they are an ideal complement to subjective image-based screening. The sensitivity of image-based BC screening might be greatly aided through the incorporation of molecular diagnostics, such as serum protein biomarkers (SPB) [21] and tumor associated autoantibodies (TAAb) [22,23].

This study evaluated the independent and combined contributions of SPB and TAAb in identifying BC using pre-biopsy blood samples collected at a single site over a six-year period from women with later known clinical outcomes. The use of pre-biopsy samples is important because: (1) it represents the intended use population when evaluating a test to be used in conjunction with inconclusive imaging results and (2) the inflammation and wound healing that occurs following a biopsy could confound serum biomarker data, potentially producing false positives or false negatives [24,25]. To our knowledge, this is the first study to directly evaluate the independent and combined contributions of SPB and TAAb in detecting BC in pre-biopsy specimens obtained from an independent cohort with known clinical outcomes.

## Materials and Methods

### Sample Collection and Serum Preparation

All samples used in this study were collected by Mercy Women’s Center-Oklahoma City under a protocol approved by the Mercy Hospital-OKC Institutional Review Board. Following written informed consent, one tube of blood was collected in a Vacutainer clot tube. Samples were drawn from two groups of patients–“healthy” controls with no evidence of breast disease (with breast MRI confirming normalcy in nearly, but not all, cases) and women scheduled to undergo a biopsy; all samples were drawn prior to biopsy. Blood was allowed to coagulate for 1.5–2 hours, then placed in a centrifuge and spun at 1,100 x *g* for 20 minutes. Samples were then further processed only if non-hemolyzed, non-lipemic, and non-icteric. Immediately after centrifugation was completed, 1 mL serum aliquots were transferred into 2 mL cryovials. All patient information was de-identified prior to distribution of specimens to Provista Diagnostics, Inc. for biomarker testing; tubes were labeled with a specimen ID number and date. Upon receipt by Provista, cryovials were placed immediately into -80°C for storage. De-identified patient demographical data was provided to Provista Diagnostics, Inc.

### Patient Population

All subjects were enrolled after imaging assessment, which may include screening mammography, diagnostic mammography, ultrasound, MRI, or any combination thereof. BI-RADS information for all subjects was obtained from the annual screening or initial visit radiology reports, even in cases where subsequent imaging resulted in a change in BI-RADS assessment.

Of the samples obtained for this study, 18 were collected from women with no evidence of breast disease (ND, no biopsy), 102 were collected from women with BBD (confirmed by biopsy), and 90 were collected from women with BC (confirmed by biopsy) (Table 1). Of these 210 samples, one was subsequently determined to be from a patient with prior BC; this sample was excluded from testing. Of the remaining 209 samples tested, 17 did not have valid measurements for one or more SPB; these samples were omitted from SPB modeling.

**Table 1:** Patient Characteristics. A total of 210 patients provided serum specimens for this study. Screening BI-RADS categories are shown for all samples and the breakdown of ND, BBD, and BC samples within each BI-RADS category are shown in parentheses. The number of samples excluded and the reason for exclusion is provided in parentheses in the “Diagnosis” section. BBD and BC were confirmed by biopsy, and all serum was obtained prior to biopsy.

	n =
All Samples	210
**Screening BI-RADS**	
**0**	3
**ND**	(0)
**BBD**	(3)
**BC**	(0)
**1**	2
**ND**	(1)
**BBD**	(0)
**BC**	(1)
**2**	29
**ND**	(17)
**BBD**	(9)
**BC**	(3)
**3**	9
**ND**	(0)
**BBD**	(9)
**BC**	(0)
**4**	96
**ND**	(0)
**BBD**	(68)
**BC**	(28)
**5**	71
**ND**	(0)
**BBD**	(3)
**BC**	(68)
**Diagnosis**	
**ND: No Evidence of Breast Disease**	18
**(Prior BC)**	(1)
**(Incomplete SPB Data)**	(1)
**BBD: Benign Breast Disease**	92
**(Incomplete SPB Data)**	(5)
**BC: Breast Cancer**	100
**(Incomplete SPB Data)**	(11)

### Serum Protein Biomarker (SPB) Measurements

Serum Protein Biomarkers selected for evaluation in this study were based on assessment of previously published literature on potential BC biomarkers [26–32]. Additional SPB known to participate in cancer-related pathways (e.g., angiogenesis and inflammation) were also included [33,34]. All SPB concentrations were determined using electrochemiluminescent (ECL)-based ELISA kits manufactured by Meso Scale Discovery (Rockville, MD). All assays were performed according to manufacturer’s specifications. Briefly, ELISA plates were blocked and protein standards and controls of known analyte concentrations were added in duplicate. Primary antibodies were diluted according to manufacturer specifications and added to all wells. Secondary antibodies, containing an ECL tag, were diluted as specified and added to all wells. All plates were analyzed using a MSD S-600 imager. To be deemed valid for SPB assessment, a sample’s result had to fall within the standard curve and have a duplicate coefficient of variation (CV) value below 20%. Any samples with duplicate measurements outside of this range were re-tested. If the re-tested values also deviated from the standard curve, the specimen was excluded from SPB analysis.

### Tumor-Associated Autoantibody (TAAb) Measurements

Tumor associated autoantibodies selected for evaluation were previously identified as potential BC discriminators in published studies [22,23]. Target proteins were produced using a one-step human coupled *in vitro* transcription translation (IVTT) kit (Thermo; Rockford, IL). All target cDNA sequences were previously inserted in the pANT7-cGST vector [23]. The target protein was purified using glutathione columns (GE Healthcare; Pittsburgh, PA) and quantified using the Qubit protein assay (Life Technologies; Grand Island, NY). A total of 40 ng of target protein was spotted onto MSD multiplex plate, and each multiplex contained a Glutathione Sepharose Transferase (GST) spot as a negative control. Assay workflow followed a standard indirect ELISA protocol. In brief, samples and plates were first blocked using Milk Diluent/Blocking Concentrate (KPL, Inc.; Gaithersburg, MD). Blocked samples were added to the plate in duplicate wells and incubated with shaking. Diluted anti-human CH2 (Pierce; Rockford, IL) was added to each well, followed by diluted SULFO tagged anti-mouse antibody (MSD). All plates were analyzed using a MSD S-600 imager. To be deemed valid for TAAb assessment, a sample had to have a duplicate CV value below 20%. Any samples with duplicate measurements outside of this range were re-tested. If the re-tested values deviated, the sample was excluded from TAAb analysis.

### Statistical Analysis

All analyses were conducted using SAS version 9.4 (SAS, Cary, NC). All provided *p*-values were two-sided and were considered significant at p < 0.05. Analyses were not adjusted for multiple comparisons due to the impact on univariate associations. Descriptive statistics were evaluated for all clinical variables between groups. For continuous variables, mean, standard deviation, median, minimum, and maximum were summarized, and statistical significance was evaluated using t-tests or Wilcoxon Rank Sum tests where applicable. For categorical variables, counts and percentages were analyzed for group differences using chi-square tests.

Autoantibody mean fluorescence intensity (MFI) was normalized using sample background MFI obtained from the GST spots. Univariable logistic regressions were used to assess the relationship between all SPB, clinical variables, and TAAb with the clinical outcome. Summaries of sensitivity, specificity, NPV, and PPV were conducted for all SPB and TAAb. All predictors that were statistically significant (univariably) were selected for inclusion into the final adjusted model. Tests for co-linearity were conducted to remove extraneous variables. An adjusted model using a combination of predictor types was selected using backward selection and an alpha = 0.15 threshold. All models were assessed using AUROC as well as evaluated for clinical sensitivity, clinical specificity, negative predictive value (NPV) and positive predictive value (PPV) using biopsy-confirmed BC or BBD as the comparator. AUC comparisons were performed using a nonparametric approach [35].

## Results

### Patient Characteristics and Biomarker Selection

The characteristics for the patients that provided pre-biopsy serum specimens for this study are provided in Table 1. There were 18 specimens collected from women with no evidence of breast disease on imaging (ND), 92 specimens collected from women diagnosed with BBD using biopsy, and 100 specimens collected from women diagnosed with BC using biopsy. BI-RADS status was collected for study participants; there was representation across all BI-RADS categories in this study. One of the patients originally assigned to the ND group was subsequently determined to have had a prior BC diagnosis; the serum obtained from this patient was excluded from testing.

A list of SPB and TAAb used in this study are provided in Table 2. Of the 209 samples tested for SPB, 17 did not have valid measurements for one or more SPB; these samples were omitted from SPB modeling (Table 1).

**Table 2:** Serum Protein Biomarkers and Tumor associated autoantibodies evaluated. *P*-values are provided for each analyte in comparing non-BC (ND and BBD) to BC (two-tailed T-test assuming heteroscedastic variance). Glutathione S-transferase (GST) was used to normalize the TAAb ratio; the *p*-value in parentheses refers to the mean GST MFI across all plates.

Biomarker Name	Biomarker Type	P-value
Cancer antigen 15.3 (CA15.3)	SPB	0.07
Cancer antigen 125 (CA-125)	SPB	0.88
Osteopontin (OPN)	SPB	0.02
Fas Ligand (FasL)	SPB	<0.01
Tumor necrosis factor alpha (TNFα)	SPB	0.04
Human epidermal growth factor receptor 2 (ErbB2)	SPB	0.06
Interleukin-6 (IL-6)	SPB	0.12
Interferon gamma (IFNγ)	SPB	0.17
Interleukin 10 (IL-10)	SPB	0.25
Interleukin 1-beta (IL-1b)	SPB	0.52
Interleukin 2 (IL-2)	SPB	0.41
Interleukin 8 (IL-8)	SPB	0.38
Carcinoembryonic antigen (CEA)	SPB	0.03
Interleukin 12 (IL-12)	SPB	0.03
Hepatocyte growth factor (HGF)	SPB	0.04
Vascular endothelial growth factor (VEGF)	SPB	0.68
Vascular endothelial growth factor subtype C (VEGF-C)	SPB	0.24
Vascular endothelial growth factor subtype D (VEGF-D)	SPB	0.01
Basic fibroblast growth factor (bFGF)	SPB	0.16
Placental Growth Factor (PIGF)	SPB	0.43
Vascular endothelial growth factor receptor 1 (FLT-1)	SPB	0.71
Angiopoietin-1 receptor (TIE-2)	SPB	0.50
Alpha-1,2-Glucosyltransferase (ALG10)	TAAb	0.55
Activating Transcription Factor 3 (ATF3)	TAAb	0.09
ATPase, H+ Transporting, Lysosomal Accessory Protein 1 (ATP6AP1)	TAAb	0.10
HLA-B-Associated Transcript 4 (BAT4)	TAAb	0.35
Brain-Derived Neurotrophic Factor (BDNF)	TAAb	0.20
BTK-Like On X Chromosome1 (BMX)	TAAb	0.94
Normal Mucosa Of Esophagus Specific (NMES1)	TAAb	0.70
Casein Kinase 1, Epsilon (CSNK1E)	TAAb	0.43
C-Terminal Binding Protein 1 (CTBP1)	TAAb	0.47
Dihydrolipoamide Branched Chain Transacylase E2 (DBT)	TAAb	0.44
Eukaryotic Translation Initiation Factor 3, Subunit E (EIF3E)	TAAb	0.35
Fibroblast Growth Factor Receptor Substrate 3 (FRS3)	TAAb	0.78
G Protein-Coupled Receptor 157 (GPR157)	TAAb	0.65
Homeobox D1 (HOXD1)	TAAb	0.80
Myozenin 2 (MYOZ2)	TAAb	0.83
Tumor Protein 53 (p53)	TAAb	0.40
Programmed Cell Death 6 Interacting Protein (PDCD6IP)	TAAb	0.39
RAS-Associated Protein RAB5A (RAB5A)	TAAb	0.19
Ras-Related C3 Botulinum Toxin Substrate 3 (RAC3)	TAAb	0.13
Selectin L (SELL)	TAAb	0.41
Collagen Binding Protein 1 (SERPINH1)	TAAb	0.23
Splicing Factor 3a, Subunit 1 (SF3A1)	TAAb	0.02
Solute Carrier Family 33 (Acetyl-CoA Transporter), Member 1 (SLC33A1)	TAAb	0.98
Sex Determining Region Y-Box 2 (SOX2)	TAAb	0.01
Transcription Factor CP2 (TFCP2)	TAAb	0.73
Tripartite Motif Containing 32 (TRIM32)	TAAb	0.45
Ubiquitin Associated Protein 1 (UBAP1)	TAAb	0.78
Zinc Finger, MYM-Type 6 (ZMYM6)	TAAb	0.88
Zinc Finger Protein 510 (ZNF510)	TAAb	0.06
		
Glutathione S-Transferase (GST)	TAAb Control	(<0.01)

### Univariate and Multivariate Analyses of Serum Protein Biomarkers (SPB)

**Fig 1:**
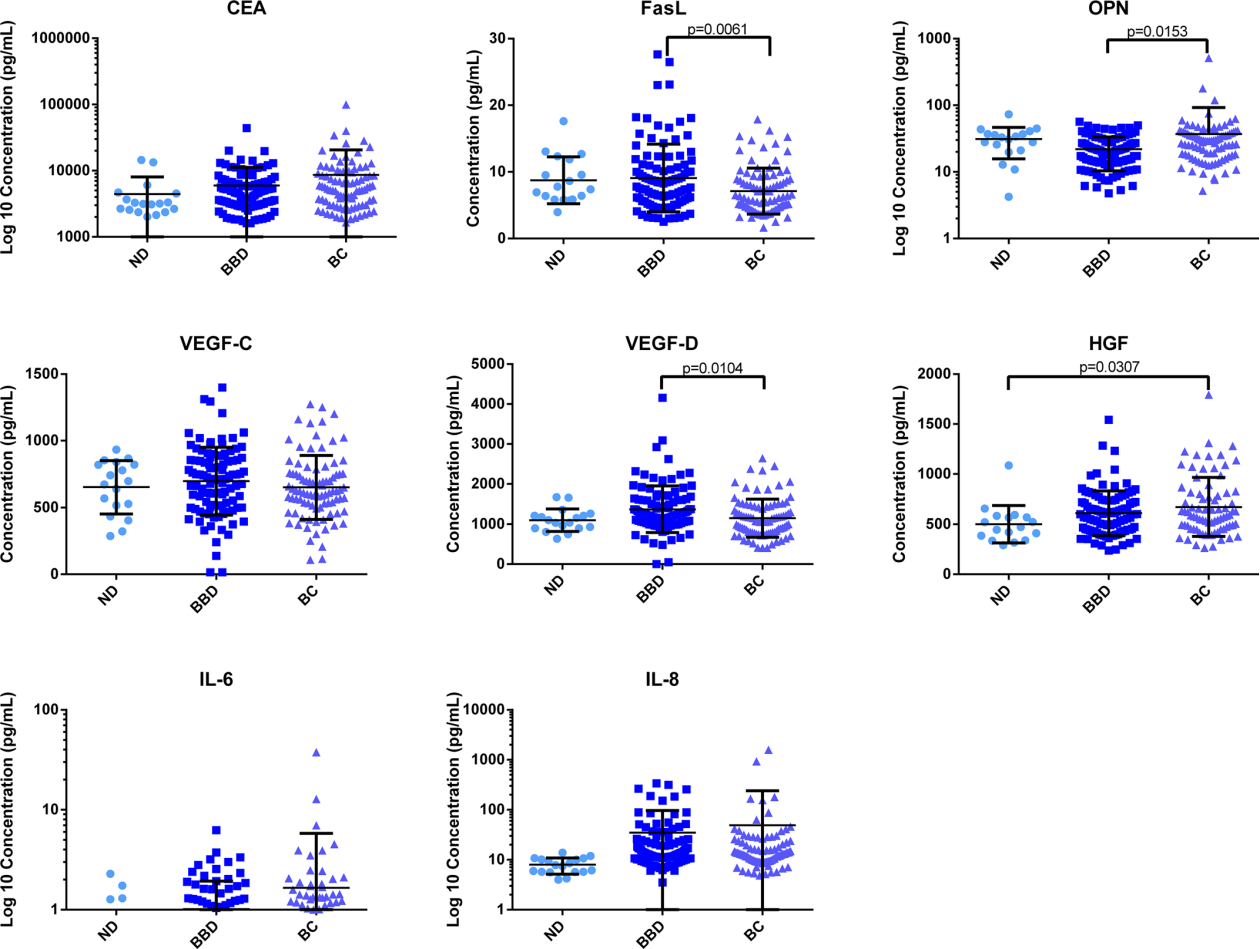
SPB Expression across Patient Groups. Shown is the comparison of no disease (ND), benign breast disease (BBD), and breast cancer (BC) samples. Graphs may be shown with y-axis represented as concentration (in pg/mL) or Log 10 concentration in order to better view analytes with wide distributions in the study population.

All 22 SPB were analyzed using an ECL-based ELISA as described in the Materials and Methods. Individual patient characteristics (including but not limited to age and family history) and clinical outcomes were extracted from each de-identified patient record. Univariate analyses were completed to determine whether SPB differed between women diagnosed with BC and women diagnosed with BBD, both of which were diagnosed using biopsy. Several SPB (OPN, FasL, TNFα, CEA, IL-12, HGF, and VEGF-D) were found to be differentially expressed at statistically significant levels (p<0.05, Table 2). Representative SPB scatterplots are provided (Fig 1).

**Fig 2:**
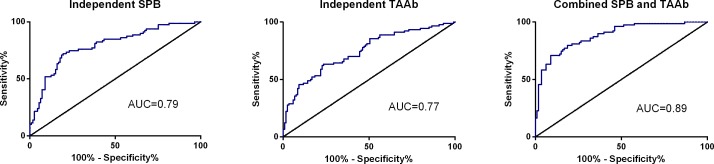
ROC curves for Models. Area under the curve (AUC) shown for each model in the inset.

To determine a panel of SPB that best differentiates BC from non-BC, a logistic regression model was created using backwards selection and an alpha = 0.15 threshold. The optimal independent SPB model demonstrated a clinical sensitivity of 74.7% and a clinical specificity of 77.0% (Table 3). The AUC for this model was 0.79 (Fig 2). It should be noted that future marker sets will need to limit the intended use population to further refine the ability of this test to distinguish cancer from non-cancer (BBD) in a controlled population.

**Table 3:** Summary of Model Performance. The biomarkers relevant to each model are given, along with model performance parameters.

Model	Biomarkers	Sensitivity	Specificity	NPV	PPV	AUC
Independent SPB	CEA, FASL, OPN, VEGFC, VEGFD, HGF	74.7%	77.0%	81.3%	69.4%	0.79
Independent TAAb	FRS3, RAC3, HOXD1, GPR157, ZMYM6, EIF3E, CSNK1E, ZNF510, BMX, SF3A1, SOX2	72.2%	70.8%	78.4%	63.3%	0.77
Combined SPB and TAAb	FASL, IL6, IL8, OPN, VEGFD, HGF, FRS3, MYOZ2, RAC3GPR157, ZMYM6, EIF3E, CSNK1E, ZNF510, BMXSF3A1, SOX2	81.0%	78.8%	85.6%	72.7%	0.89

### Univariate and Multivariate Analyses of Tumor-associated Autoantibodies (TAAb)

**Fig 3:**
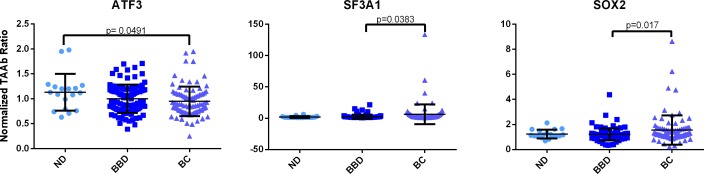
TAAb Expression across Patient Groups. Shown is comparison of no disease (ND), benign breast disease (BBD), breast cancer (BC). Graphs are shown with y-axis represented as normalized TAAb ratio, wherein the target signal is normalized against sample background.

Tumor associated autoantibody expression was measured for all targets using ECL indirect ELISA, as described in the Materials and Methods. Univariate analyses were completed to determine whether TAAb differed between women diagnosed with BC and women diagnosed with BBD, both of which were diagnosed using biopsy. There were two TAAb target ratios that were statistically significant (SF3A1 and SOX2, Table 2). It should be noted that sample background levels (represented by GST MFI) in this study were significantly (p<0.01) higher in specimens from non-BC patients as compared to BC patients. Median sample background was 1,679 for BC samples and 2,302 for non-BC samples (data not shown). High sample background tends to result in lower TAAb ratios because ratios are normalized to sample background MFI, thus it is possible that a subset of non-BC sample TAAb ratios were aberrantly low. This background could be due to the IVTT platform used to evaluate TAAb expression in this study. Representative TAAb scatterplots are provided (Fig 3).

To determine a panel of TAAb that best differentiates BC from non-BC, a logistic regression model was created using backwards selection and an alpha = 0.15 threshold. The optimal independent TAAb model demonstrated a clinical sensitivity of 72.2% and a clinical specificity of 70.8% (Table 3). The AUC for this model was 0.77 (Fig 2), which was comparable to the independent SPB model performance.

### Multivariate Analysis of Combination of SPB and TAAb

The performances of models involving SPB or TAAb alone were modest (AUC of 0.79 and 0.77, respectively). It was hypothesized that test performance may be improved by combining TAAb and SPB into a single model, i.e., applying a combinatorial approach. To determine a combination of SPB and TAAb that best differentiates BC from non-BC, all possible markers were included in a logistic regression model and the strategy of using backwards selection and a threshold of 0.15 was applied to identify a combinatorial model. The combination of SPB and TAAb improved clinical sensitivity and clinical specificity to 81.0% and 78.8%, respectively (Table 3), which were both superior to that of the independent SPB and TAAb models. Furthermore, NPV and PPV were highest for this combined model. The AUC of this combined model was 0.89 (Fig 2), a significant improvement over AUCs for TAAb alone (p<0.0001) and SPB alone (p = 0.0012). These results suggest that application of a model that combines SPB and TAAb data can identify the presence of BC from a pre-biopsy serum sample with high sensitivity and specificity.

Samples from participants who were assessed as BI-RADS 5 were included to provide a greater number of subjects diagnosed with breast cancer. However, a biomarker test would not greatly impact SOC in these patients, who would be recommended for biopsy regardless of biomarker testing. Table 4 breaks down the performance of each model based on subject BI-RADS. While sensitivity and PPV in the BI-RADS 5 population were generally better than that seen in indeterminate subjects (BI-RADS 3/4), this is likely due to the reduced breast cancer prevalence in the BI-RADS 3/4 population relative to the BI-RADS 5 population. Conversely, specificity and NPV in the BI-RADS 3/4 population were generally better than that seen in the BI-RADS 5 population, which is likely due to the larger number of BBD subjects in the BI-RADS 3/4 population (only 3 participants in the BI-RADS 5 category were diagnosed as BBD). Overall, the data shown in Table 4 support the conclusion that a model combining SPB and TAAb is superior to models that utilize only SPB or TAAb in detecting breast cancer. It should be noted, however, that this is a test population and these values could vary in different intended use populations.

**Table 4:** Summary of Model Performance based on subject BI-RADS. Model performance is shown for subjects categorized as BI-RADS 3 or 4 and BI-RADS 5. BC prevalence is shown to indicate the number of subjects diagnosed with breast cancer as a percentage of the total for each BI-RADS population. Some samples that were excluded from SPB and Combined model building due to incomplete SPB data.

**Independent TAAb**	**BC Prevalence**	**Sensitivity**	**Specificity**	**NPV**	**PPV**
BI-RADS 3/4	26.67%	57.14%	68.83%	81.54%	40.00%
BI-RADS 5	95.77%	67.65%	0.00%	0.00%	93.88%
**Independent SPB**	**BC Prevalence**	**Sensitivity**	**Specificity**	**NPV**	**PPV**
BI-RADS 3/4	25.51%	64.00%	82.19%	86.96%	55.17%
BI-RADS 5	95.24%	75.00%	100.00%	16.67%	100.00%
**Combined SPB/TAAb**	**BC Prevalence**	**Sensitivity**	**Specificity**	**NPV**	**PPV**
BI-RADS 3/4	25.51%	72.00%	86.30%	90.00%	64.29%
BI-RADS 5	95.24%	80.00%	33.33%	7.69%	96.00%

Independent models were created to be inclusive of covariables, such as age and BI-RADS (S1 Table, S1 Fig). These models did demonstrate a slight improvement in performance, but also may have been more vulnerable to confounding factors relating to subject demographics. Importantly, the age & BI-RADS-inclusive models also demonstrate improved performance in the combined model compared to SPB or TAAb alone models.

## Discussion

In clinical practice, pathological confirmation (biopsy) is necessary to determine whether an abnormality noted on imaging is benign or malignant. The limited ability of an image to inform diagnosis of tissue without a physical tissue specimen is the impetus for developing a serum-based assay that detects the biochemical cues of the presence of BC to further elucidate the nature of an abnormality noted on an image.

In this study, independent SPB and TAAb models demonstrated clinical sensitivities of 74.7% and 72.2%, respectively, and clinical specificities of 77.0% and 70.8%, respectively (Table 3). The clinical sensitivity and clinical specificity of the combinatorial model was 81.0% and 78.8%, respectively (Table 3), which was superior to either independent model. The overall performance of the combinatorial model was superior to either independent model; the AUCs were comparable for SPB and TAAb independent models (0.79 and 0.77, respectively), whereas the AUC for the combinatorial model was 0.89 (Fig 2). These data support utilizing a combined SPB and TAAb approach to identify BC in women with suspicious imaging findings. It is critical to note, however, that these are proof-of-concept studies and the application to a specific intended use population may yield different results.

The high NPV (85.6%, Table 3) in such a high prevalence population indicates that, clinically, a negative serum assay result may guide close observation of many of the abnormalities noted on imaging that otherwise would have been biopsied. While these data are promising, it should be noted all samples in this study were collected from a single source, which can result in a geographical bias; however, the benefit of this study design is that it enables the direct assessment of the contribution of SPB and TAAb to diagnostic identification of BC in a defined patient cohort where pre-analytical variables and clinical outcome data collection differences should be minimized, allowing an accurate assessment of the true contribution of these biomarkers as independent and combined entities. Different blood collection techniques, serum isolation procedures, and storage at multiple sites could affect the expression of these biomarkers, confounding results. Large prospective, randomized, multi-site blinded clinical trials are currently underway to further understand the contribution of SPB and TAAb tests to identifying BC, specifically in the BI-RADS 3 and/or 4 patient populations. The results of these trials will further elucidate the clinical utility of SPB and TAAb combinatorial protein biomarker assays to aid in the detection of early BC and to guide decisions between imaging and tissue biopsy.

## Supporting Information

S1 Fig ROC curves for Age & BI-RADS-Inclusive Models. Area under the curve (AUC) shown for each model in the inset.
(TIF)

S1 Table Performance of models utilizing subject age. P-values are shown for the comparisons of Combined vs. Independent TAAb and Combined vs. Independent SPB
(DOCX)

S2 Table Raw data for all samples and analytes. (XLSX)
