# Supplementary material for: Integration of Serum Protein Biomarker and Tumor Associated Autoantibody Expression Data Increases the Ability of a Blood-Based Proteomic Assay to Identify Breast Cancer
Source: PLoS One. 2016 Aug 10;11(8):e0157692. doi: 10.1371/journal.pone.0157692 (PMC4980010; doi:10.1371/journal.pone.0157692)
Supplement: S1 Table — P-values are shown for the comparisons of Combined vs. Independent TAAb and Combined vs. Independent SPB (DOCX) [file pone.0157692.s002.docx]

| **Age & BI-RADS- Inclusive Model** | **Biomarkers** | **AUC** | **p-value** |
| --- | --- | --- | --- |
| Independent SPB | Age, BI-RADS, FRS3, RAC3, HOXD1, GPR157, ZMYM6, EIF3E, CSNK1E, ZNF510, BMX, SF3A1, SOX2 | 0.88 |  |
| Independent TAAb | Age, BI-RADS, CEA, FASL, OPN, VEGFC, VEGFD, HGF | 0.89 |  |
| Combined SPB and TAAb | Age, BI-RADS, FASL, IL6, IL8, OPN, VEGFD, HGF, FRS3, MYOZ2, RAC3, GPR157, ZMYM6, EIF3E, CSNK1E, ZNF510, BMX, SF3A1, SOX2 | 0.93 | **SPB: 0.024** |
|  |  |  | **TAAb: 0.001** |
